# Silvicultural Practices for Diversity Conservation and Invasive Species Suppression in Forest Ecosystems of the Bundala National Park, Sri Lanka

**DOI:** 10.3390/plants13010121

**Published:** 2023-12-31

**Authors:** Channa Suraweera, Josef Gallo, Zdeněk Vacek, Jan Cukor, Stanislav Vacek, Martin Baláš

**Affiliations:** 1 Faculty of Forestry and Wood Sciences, Czech University of Life Sciences Prague, Kamýcká 129, Suchdol, 165 00 Prague, Czech Republic; pinidiya@fld.czu.cz (C.S.); vacekz@fld.czu.cz (Z.V.); cukor@fld.czu.cz (J.C.); vacekstanislav@fld.czu.cz (S.V.); balas@fld.czu.cz (M.B.); 2 Forestry and Game Management Research Institute, v.v.i., Strnady 136, 252 02 Jíloviště, Czech Republic

**Keywords:** biodiversity, forestry, silviculture, climate change, *Prosopis juliflora*

## Abstract

Forest ecosystems in Sri Lanka are under pressure from intensive human activity and climate change. Invasive species are one of the greatest threats to autochthonous species and ecosystems. In Bundala National Park of Sri Lanka, there are efforts to control and limit the spreading of unwanted invasive *Prosopis juliflora* (Sw.) DC. and *Opuntia dillenii* (Ker-Gawl.) Haw., which poses a significant risk to natural ecosystem conservation. Nine different treatment variants (four replications) were used to test which management approach provides the control of *Prosopis juliflora*. This research is based on nine repeated measurements from 2017 to 2021 on 36 permanent research plots (each 625 m^2^) with 27 observed plant species and a total of 90,651 recorded plant individuals. The results confirmed that the dynamics of species richness, heterogeneity, and evenness showed significant differences between treatments during the five years of dynamics. The lowest species diversity was found in the control variant, followed by treatments based on the hard pruning and thinning of *Prosopis juliflora* trees. In contrast, strategies emphasizing the complete uprooting of *Prosopis juliflora* trees, replanting, and support of the natural regeneration of native species showed high species diversity and a high overall number of plant species. Generally, treatments had a significant effect on species diversity and the number of individuals of *Prosopis juliflora*, while changes in the overall number of plant species were more affected by time and succession. Silvicultural treatments including pruning, uprooting, and thinning have proven to be essential tools for nature conservation across various sites, aimed at enhancing habitat diversity in the face of ongoing climate change.

## 1. Introduction

The global landscape has been strongly influenced by human activity in the last millennium [1,2]. Human population growth and the intensive impact on nature are considered the main drivers of natural ecosystem transformation across most of the terrestrial biosphere [3,4], causing irreversible changes in global biodiversity [5]. The direct negative impacts are related to the exploitation of nature through hunting, fishing, logging, harvesting, and mining activities [6,7,8,9]. As a result, the terrestrial biosphere has transitioned from predominately wild to mostly anthropogenic during the past 300 years [3,10,11,12].

Native forests are one of the most endangered ecosystems devastated by deforestation and forest fragmentation worldwide [13], especially in South America, Asia, and Oceania in recent decades [13,14]. At the same time, deforestation represents the greatest threat to terrestrial biodiversity in tropical regions [15,16,17,18]. Tropical rainforests are the most diverse habitats, with approximately 50% of the world’s species [19,20,21]. Almost half of these forests have either been degraded in various ways or are secondary forests created by humans [22,23] or by various natural disturbances [24]. However, native ecosystems, including natural forests, are endangered by humans in several ways. The spreading of invasive species represents one of the challenges for forest ecosystems caused by human activity [25,26,27].

Therefore, invasive alien plant species (hereafter “IAS”) are considered one of the greatest threats to global biodiversity and the sustainable functioning of natural ecosystems [27,28]. IAS can be defined as species introduced to a natural habitat whose establishment and spread threaten the ecosystem, habitat, or species with economic or environmental harm [29,30]. Therefore, they are also defined as transformer species [31]. The purposeful introduction of non-native tree species is motivated by wood production in the case of forest management and the preservation of high-quality wood production [32,33,34,35]. Such an introduction can be beneficial in terms of increased carbon sequestration, oxygen production, and species richness, as well as other biodiversity indicators [32,33]. On the other hand, in a wide range of environments, introduced fast-growing exotic tree species can become invasive and pose a serious threat to natural ecosystems by replacing the existing vegetation [33,36]. The negative effects of IAS on protected areas have been documented worldwide [37]. Sri Lanka can be used as an example of an area heavily damaged by IAS [36,38]. Sri Lanka’s tropical forests support a unique degree of biodiversity because of the area’s diverse topographical and climatic conditions, with rapidly decreasing biodiversity due to the extinction of native tropical forests [39].

Much of Sri Lanka’s original climax forest found further inland has been degraded due to former timber and fuel wood extraction and shifting cultivation [39]. Degraded areas do not revert to closed-canopy forests through natural succession but tend to develop into scrub or scrubby woodland. This degradation has led to the establishment of scrub while also providing opportunities for colonization by invasive exotic species, notably *Prosopis juliflora* (Sw.) DC. and the cactus *Opuntia dillenii* (Ker-Gawl.) Haw., that suppress the natural regeneration of indigenous species [40] (see Figure S1 in Supplementary Materials). Only a few patches of undisturbed old-growth forest remain, and protected areas (PAs) account for 26.5% of the area within the country.

At present, there are 26 national parks covered by the Fauna and Flora Protection Ordinance. Bundala National Park (BNP), one of the most important national parks in Sri Lanka, was chosen for biodiversity research [41,42] and further management measures to mitigate the spread of introduced plant species. The main native tree species that characterizes BNP is *Manilkara hexandra* (Roxb.) Dubard. [42], which is now threatened by introduced plant species, particularly *Opuntia dillenii* and *Prosopis juliflora*, both originating in Central and South America [43], which are spreading on degraded sites in fragments of original ecosystems in about 17% of the national park area [40,44]. These introduced species tend to spread further and damage native biodiversity [45]. They are considered an increasing problem, as they substantially limit the occurrence of native plant species [46]. From this point of view, for example, Yang et al. [47] and Alford et al. [48] showed that the secondary metabolites released by invasive plants directly inhibit the seed germination of native plants while indirectly promoting the growth of introduced species through different processes in nutrient cycling. Similarly, Shipunov et al. [49] reported that after a host invasion, endophytes can increase their competitiveness by producing metabolites inhibiting the evolutionarily native plants. This opinion is also supported by Aschehoug et al. [50]. Effective species management requires applications to enhance the functionality of native plant species and limit non-native introduced plants, especially *Prosopis juliflora* and *Opuntia dillenii* [51,52]. *Prosopis juliflora* forms dense, impenetrable, monospecific thickets, impairs the growth of grasses, and reduces the overall biodiversity of the area by decreasing species abundance, species distribution, and ecosystem functions [53].

*Prosopis juliflora* was introduced to Sri Lanka by the Forest Department for reforestation purposes, and presently, a massive area of BNP has been invaded by it, which results in heavy damage to BNP as a refuge for diverse flora and fauna [54]. This fast-growing, arid, and saline-soil-tolerant evergreen tree is native to South America, Central America, and the Caribbean [55]. Concern about deforestation, desertification, and fuel wood shortages in the 1970s and 1980s prompted a wave of projects that introduced *Prosopis juliflora* and other very resistant tree species to new environments worldwide, where they shortly became a considerable nuisance.

*Opuntia dillenii* is a succulent, pricky pear shrub species from the tropical Americas and naturalized in many other parts of the world [56]. In India and Sri Lanka, it is considered an invasive species. After the initial rapid spread of the invasive species and the subsequent disturbance of coastal vegetation [57], it was shown that it could be suppressed by its natural enemies [58]. Therefore, the focus gradually shifted to *P. juliflora*, which occupies the most important upper tree layer yet shows no sensitivity to pests and diseases. According to the last baseline survey conducted by the Department of Wildlife Conservation (DWC), *Prosopis juliflora* and *Opuntia dillenii* covered approximately 486 ha (8%) and 567 ha (9%) in the total area of the park, respectively [44]; however, these two invasive species are spreading in BNP, which requires active management in the form of silvicultural treatments.

Previous studies found that secondary vegetation can, in some cases, support high biodiversity [59,60,61,62], but in other cases, the opposite was shown [63,64,65]. The question is what the situation is in Sri Lanka, where even forests in national parks have been greatly affected by humans and natural disturbances in the past [66,67]. At the same time, the conservation value of ecosystems, structural complexity, and diversity is critical [68,69,70,71,72]. The main challenge of conservation management is to mitigate the negative impacts of IAS on natural ecosystems and to stop the spreading of particular invasive tree species. Therefore, the main aims of this study are to evaluate (i) the number of plant species that occur in the area of interest; (ii) the species diversity (richness, heterogeneity, and evenness) of the forest ecosystem; and (iii) different silvicultural treatments applied to regulate the main invasive non-native species *Prosopis juliflora* in the forest ecosystem in Bundala National Park during 2017–2021.

## 2. Results

### 2.1. Number of Plant Species and Individuals

In 2017–2021, 27 observed plant species with a total of 90,651 plant individuals were recorded on 36 permanent research plots. The aggressive species *Opuntia dillenii*, with 66%, reached the highest representation in plant species composition, followed by invasive *Prosopis juliflora* (7%), *Stachytarpheta indica* (L.) Vahl (5%), *Solanum melongena* L. (4%), and *Senna auriculata* (L.) Roxb. (4%). Other plant species had shares in the plant species composition of less than 3%, with the lowest representation (>0.1%) by *Manilkara hexandra*, *Derris* spp., and *Limonia acidissima* (L.). According to the treatments, variants T5–8 showed a significant (*p* < 0.05) positive effect on the elimination of *Prosopis juliflora* individuals in 2017–2021 (Table 1). During the same period, there was also a significant (*p* < 0.05) decline in *Opuntia dilleniid* individuals, but this was caused by the insect *Dactylopius opuntiae* (Cockerell). On the other hand, the hard pruning (T1, T2) and thinning (T3, T4) of *Prosopis juliflora* had no significant (*p* > 0.05) effect on the number of individuals of either invasive plant species compared to the control variant (T9). However, the complete uprooting of *P. juliflora* to allow for natural regeneration (T5) had the highest positive effect on the number of individuals of different plant species, especially *Salvadora persica* L., *Bauhinia racemosa* Lam., *Flueggea leucopyrus* Willd, *Solanum melongena*, *Stachytarpheta indica*, *Senna tora* Roxb., *Achyranthes aspera* L., and *Cassia fistula* L.

Over time, a significant change in the representation of individual species was observed (Table 2). During the four-year study (nine repeated measurements—Table 4, the largest decrease in the number of plant individuals was recorded for *Opuntia dillenii* (with a decrease of 900–180,800% depending on the treatment). Generally, the number of plant individuals was observed to decrease in the case of *Tamarindus indicus* L., *Pongamia pinnata* L., *Achyranthes sapera*, *Madhuca longifolia* (J.Koenig ex L.) J.F.Macbr., and *Schleichera oleosa* (Lour.) Oken. A predominant increase in representation was observed for all other plant species (including *Prosopis juliflora*), especially *Terminalia arjuna* (Roxb.) Wight & Arn., *Phyllanthus emblica* L., *Drypetes sepiaria* (Wight & Arn.) Pax & K.Hoffm., and *Lantana camara* L. Regarding *Prosopis juliflora*, hard-pruning variants had the best results in preventing its invasive spread compared to the low effect of thinning (T3, T4) and complete uprooting of *P. juliflora* trees (T5, T6).

### 2.2. Species Diversity

**Figure 1:**
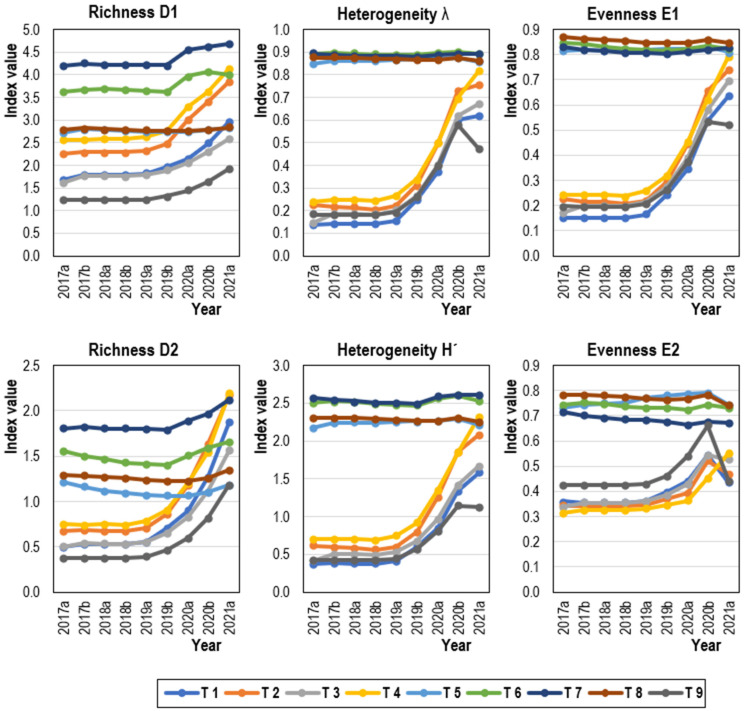
The dynamics of species diversity (richness, heterogeneity, and evenness) differentiated according to the treatment (T1–T9) in 2017–2021.

The dynamics of species diversity (richness, heterogeneity, and evenness) showed differences between treatments in 2017–2021 (Figure 1). Over time, all studied species indices rapidly increased after 2019 in the case of hard-pruning (T1, T2) and thinning (T3, T4) treatments of *Prosopis juliflora*, together with the control variant. On the other hand, only minor changes (even a decline in species evenness) were observed for variants T5–8. Overall, the significantly (*p* < 0.05) highest and very rich species diversity and the largest number of plant species (23×) were observed in two variants: the replanting of the chosen indigenous species on sites free of *Prosopis juliflora* (T7), followed by the complete uprooting of *Prosopis juliflora* trees and the replanting of the chosen indigenous species (T6, 21 plant species; Table 3). On the other hand, the lowest diversity (after control variant T9) was observed in T1 and T3 with 12 plant species.

### 2.3. Interaction among Species Diversity, Plant Density, and Treatments over Time

**Figure 2:**
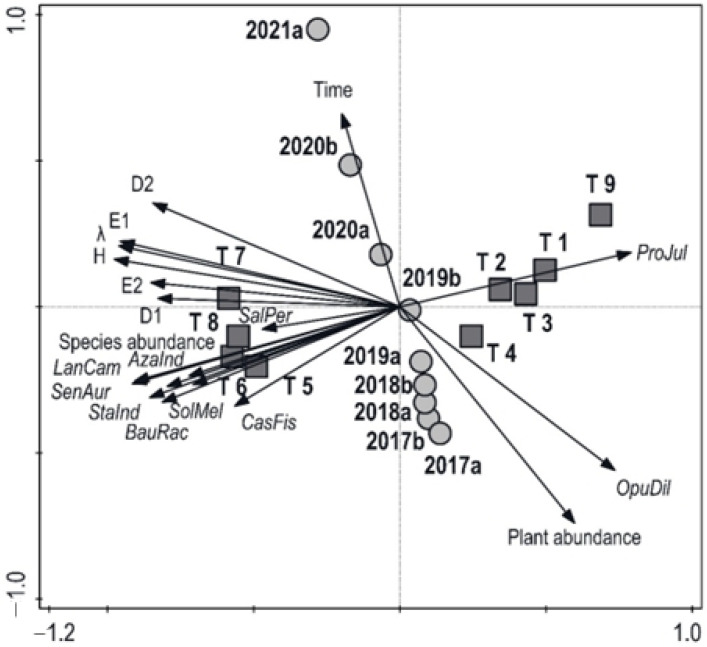
Ordination diagram showing the results of principal component analysis of the relationships between species diversity (D1 and D2—species richness; *λ* and *H*′—species heterogeneity; E1 and E2—species evenness), number of individuals of ten selected most frequent plant species (*ProJul*—*Prosopis juliflora*; *OpuDil*—*Opuntia dillenii*; *CasFis*—*Cassia fistula*; *SolMel*—*Solanum melongena*; *BauRac*—*Bauhinia racemosa*; *StaInd*—*Stachytarpheta indica*; *SenAur*—*Senna auriculata*; *LanCam*—*Lantana camara*; *AzaInd*—*Azadirachta indica*; *SalPer*—*Salvadora persica*), overall plant abundance (total number of plant individuals), species abundance (number of plant species), ● time period (2017a–2021a), and ■ treatments (T1–T9).

The results of the principal component analysis expressing the relationships between structure, diversity, production, and individual variants are presented in the form of an ordination diagram in Figure 2. The first ordination axis represents 61.75%, the first two axes represent 73.57%, and the four axes together account for 85.12% of the data variability. The x-axis represents species richness (D1) and species evenness (E2). The y-axis represents time dynamics from 2017 to 2021. Over time, the overall number of plant individuals decreased, along with the number of individuals of the most frequent species, *Opuntia dillenii*, while species diversity increased, especially species diversity D2. The number of individuals of *Prosopis juliflora* was negatively correlated with the number of plant species and the number of individuals of other plant species (except *Opuntia dillenii*). The lowest explanatory variable in the ordination diagram was the number of individuals of *Salvadora persica*. The lowest species diversity was found in the control variant (T9), followed by variants focused on the hard pruning (T1, T2) and thinning (T3, T4) of *Prosopis juliflora* trees, while variants T5–8 showed high diversity and a high number of individuals of other plant species. Generally, treatments had a substantial effect on species diversity and the number of individuals of *Prosopis juliflora*, while the overall number of plant species was more affected by time.

## 3. Discussion

This study confirmed the invasive behavior of both evaluated non-native species, while the most aggressive species, *Opuntia dillenii*, reached the highest representation in the plant species composition (66.2%). After the initial rapid spread of the invasive species and the subsequent disturbance of coastal vegetation [57], it was shown that it could be suppressed by its natural enemy, the cochineal insect (*Dactylopius opuntiae* (Cockerell, 1929)), which is used as an effective bio-control measure [58]. Therefore, the focus gradually shifted to *P. juliflora*, which occupies the most important upper tree layer and shows no sensitivity to available bio-control measures. In our study, *Prosopis juliflora*, with 6.5% of the plant composition, was the second-most-common invasive species.

Based on the colonization of the study area by IAS, the impact of various approaches to active human management on the state of the involved alien-infested sites was evaluated. From no intervention, through thinning and hard pruning, to the most intensive measures, including complete uprooting, the effectiveness of the provided measures was evaluated by different indices of the resulting biodiversity. The number of plant species and interactions were also calculated. The overall number of plant species decreased with time and succession, together with the number of individuals of *Opuntia dillenii* (as presented in Figure 2). The decline in *Opuntia dillenii* was caused by a natural enemy, *Dactylopius opuntiae* [58]. The number of individuals of *Prosopis juliflora* was negatively correlated with the number of native plant species. *Prosopis juliflora* showed resistance to most of the realized measures. Hard-pruning variants showed the best results in suppressing *Prosopis juliflora*, while thinning and complete uprooting had less of an effect. Edirisinghe et al. [73] reported a positive effect of complete uprooting while noting the danger of reinvasion three years after the application of the measure. The ambiguous results of measures to suppress IAS are evident from the considerable distribution of these species in the area. According to the last baseline survey conducted by the Department of Wildlife Conservation (DWC), *Prosopis juliflora* and *Opuntia dillenii* covered approximately 486 ha (8%) and 567 ha (9%) of the total park area, respectively [42]. The dominant presence of both aggressive species causes a change in natural processes, making them non-functional on many sites [74].

One of the aims of the performed treatments was to promote the desired native plant species diversity by either direct planting or natural succession/regeneration. Species diversity was evaluated based on species richness, heterogeneity, and evenness (Figure 1 and Table 3). Two indices were selected for analysis from each of the three indicators, as recommended by other studies for the objective interpretation of results [75,76]. Although the objective of this study is not to compare individual diversity indices with each other, there may be differences between them. For example, the study of [77] reported that the Margalef index was the best-performing indicator that passed the assessment criteria and was better than the Menhinick index in terms of species richness.

The most desirable planted species were *Pongamia pinnata*, *Tamarindus indicus*, *Cassia fistula*, *Schleichera oleosa*, *Madhuca longifolia*, *Syzygium cumini* L., and *Vitex altissima* (L.f.). The desired herb species Achyranthes aspera was not directly planted. Bundala’s iconic *Manilkara hexandra* performed best on the sites where *Prosopis juliflora* was absent, and the native species was supported by planting. Such sites showed the highest diversity indices, including species richness, heterogeneity, and evenness. This is per the authors of [43], who suggest the presence of over-mature cohorts and the occurrence of tree cankers as other threatening factors for native species, together with the presence of *Prosopis juliflora*. Other declining native species were most notably *Achyranthes sapera*, *Pongamia pinnata*, and *Tamarindus indicus*. The species that benefited most were *Terminalia arjuna*, *Phyllanthus emblica*, and *Lantana camara*. Over time, the overall number of plants decreased, together with the number of individuals of the most frequent species, *Opuntia dillenii*, while species diversity increased, particularly species richness. The number of individuals of *Prosopis juliflora* was negatively correlated with the number of plant species and the number of plant species other than *Opuntia dillenii*. This could be due to species complementarity, as suggested by Cardinale et al. [78]. The lowest explanatory variable in the ordination diagram was the number of individuals of *Salvadora persica*. This species possesses high tolerance to different climatic and soil conditions, including saline soils, and the ability to grow even on salt marshes or sand dunes on the coasts [79]. This plant has many uses and benefits for people, such as food, medicine, and other products [80]. The lowest species diversity was found in the control variant, followed by variants based on the hard pruning and thinning of *Prosopis juliflora*. Both the control and only partial removal of *Prosopis juliflora* allowed further spreading [81]. Variants including the complete removal of the trees plus roots showed higher diversity and a higher number of native plant species, in accordance with previous studies [59]. Intensified invasion by *Prosopis juliflora* and *Opuntia dillenii* reduces hospitable areas for grass-type plant species, which are substantial food sources for grazing ungulates and wild elephants. This leads to food scarcity for megaherbivores, which ultimately leads to human vs. elephant conflicts and increased wild animal mortality due to human threats [82].

In general, further research is needed to study the allelopathic effects of those species, including their seed banks and germination capacity under harsh environmental conditions that may occur due to different climate change scenarios [83]. Managing the spread of IAS in forest ecosystems requires action from stakeholders directly involved in the conservation of forests, along with those using forests for trade, health, or tourism [27,84]. As much as *P. juliflora* is an invasive plant that causes many problems for the environment and wildlife, it may also have some potential benefits for the people who live near it. Therefore, it is essential to explore how *P. juliflora* can be used in different ways that can help the local communities. For example, *P. juliflora* may be used as a source of fuel, fodder, medicine, or honey [85,86]. However, its negative impacts still prevail. The competitive ability of these invasive plants is enhanced by the production of secondary metabolites [87]. For example, Yang et al. [47] and Alford et al. [48] demonstrated that secondary metabolites released from invasive plants directly inhibit the seed germination of native plants and indirectly support the growth of introduced species by altering nutrient cycling. Similarly, Shipunov et al. [49] and Aschehoug et al. [50] reported that in this invasion, endophytes can increase the competitiveness of non-native species by producing metabolites inhibiting evolutionarily native plants. Similar results were found in Ethiopia, where besides suppressing native species, a dense cover of *Prosopis* decreased livestock productivity [88]. In Sudan, the production of *Prosopis* biomass was further improved by hard pruning [89]. Also, in Kenya, a negative effect of *Prosopis juliflora* on native vegetation due to changes in habitat and vegetation conditions was found [90]. Though the economic impact from the establishment of these species in new areas is difficult to capture, costs well exceed USD 150 billion annually in the United States, accounting for inflation due to lost productivity and increased management [91,92].

## 4. Materials and Methods

### 4.1. Study Area

**Figure 3:**
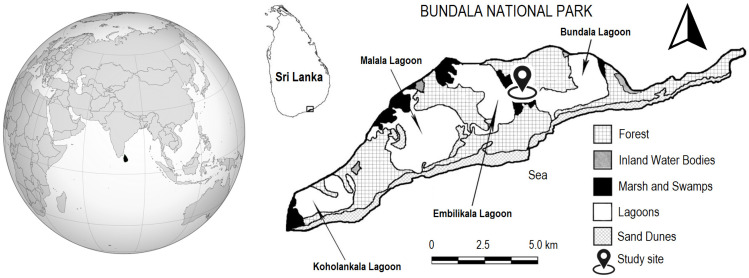
Map of Bundala National Park in Sri Lanka with four marked research blocks.

Bundala National Park (BNP) (Figure 3) consists mainly of dry, thorny scrubland and shallow brackish water lagoons with rich biodiversity. It was created primarily to protect wetlands and coastal areas in the south of Sri Lanka. A total of 383 plant species belonging to 90 families were documented, including 6 endemics and 7 species that are locally endemic [46,57,93]. BNP is among the premier bird-watching sites in Sri Lanka, with almost 200 bird species having been recorded in the national park. Therefore, Sri Lanka’s first wetland ecosystem was declared a Wetland of International Importance at the time of Sri Lanka’s 15 October 1990 ratification of the Ramsar Convention, covering an area of 6216 ha [30]. It was declared a national park in 1992 [41].

BNP lies in the Arid Zone, with a mean annual temperature of 27 °C and mean annual rainfall ranging from 900 mm to 1300 mm, with two peak periods of rainfall in April–May and October–November and an extensive intervening dry period between May and September (the Maha season and the Yala season) [93]. The elevation of the park ranges from 0 to 10 m a.s.l. with mostly flat terrain. Soils are Red Earths and sandy Regosols [52]. The vegetation of BNP is very diverse, showing a natural succession from low, creeping plants that have colonized the beach and sand dunes to climax forest, variously referred to as thorny, dry semi-evergreen, and dry-mixed evergreen forest [94,95]. Additionally, a range of vegetation types occur in the lagoons and low-lying areas, including salt marsh, mangrove, and aquatic vegetation [44,96]. Its waterfowl populations are world-renowned and best seen during the winter migratory season, as Sri Lanka lies at the southern limit of the bird flyway from Russia and China.

### 4.2. Data Collection and Treatments

For this study, silvicultural treatments such as thinning [97], pruning [98], uprooting, the replanting of native and local species, weeding, and pest control [99,100] were used. Nine different variants of silvicultural treatments (Table 4) were used for the desired control/elimination of invasive *Prosopis juliflora* (treatment was not performed on *Opuntia dillenii*). The design of the study was implemented in 25 × 25 m (625 m^2^) plots repeated in four blocks. Treatments (T1–T9) were applied separately in randomly selected plots and replicated uniformly in four blocks at the selected site (B1–B4).

According to the aforementioned treatments, we used a randomized complete block design (RCBD), which is the standard design for agricultural and ecological experiments, where similar experimental units are grouped into blocks or replicates. One month after the silvicultural treatments, monthly data for five consecutive years began to be collected. At the beginning of the experiment, all baseline data from the chosen plots were collected. The number of plant species that were present at the sites was counted and recorded separately. The density of plant species (number of individuals present per unit of ground area [101]) present at the sites was determined separately by counting individual plants. Silvicultural treatments were repeated after 1.5 years. In all, nine repeated measurements from 2017–2021 were taken on 36 permanent research plots. The following 27 plant species were classified on the plots: *Prosopis juliflora*, *Opuntia dillenii*, *Salvadora persica*, *Lantana camara*, *Senna auriculata*, *Limonia acidissima*, *Bauhinia racemosa*, *Azadirachta indica*, *Drypetes sepiaria*, *Manilkara hexandra*, *Randia dumetorrum*, *Derris* spp., *Flueggea leucopyrus*, *Solanum melongena*, *Stachytarpheta indica*, *Senna tora*, *Achyranthes aspera*, *Pongamia pinnata*, *Tamarindus indicus*, *Chloroxylon swietenia*, *Cassia fistula*, *Schleichera oleosa*, *Madhuca longifolia*, *Syzygium cumini*, *Vitex altissima*, *Terminalia arjuna*, and *Phyllanthus emblica*.

### 4.3. Data Analysis

The collected data were evaluated using appropriate statistical software to analyze the results. The number of species was counted. Biodiversity changes were evaluated using various diversity indices. The following indices were calculated: species richness according to Margalef [102] and Menhinick [103], species heterogeneity according to Simpson [104] and Shannon [105], and species evenness according to Pielou [106] and Hill [107] (Table 5).

Statistical analyses of species diversity between individual treatments were processed in Statistica 13 (13.6.0.) software (StatSoft, Tulsa, OK, USA). Data were first tested using the Shapiro–Wilk normality test and then the Bartlett variance test. When both requirements were met, the differences between the examined parameters were tested by performing a one-way analysis of variance (ANOVA), followed by the Tukey HSD test. If normality and variance requirements were not met, the investigated characteristics were tested by performing the nonparametric Kruskal–Wallis test. Multiple comparisons after the Kruskal–Wallis test were performed using the method described by Siegel and Castellan Jr. [108]. Principal component analysis (PCA) was performed in the CANOCO 5 program (Microcomputer Power) to evaluate the relations between plant species, species diversity, time, and treatment variants. Before the analysis, the data were standardized and centralized. The results of PCA were illustrated by an ordination diagram.

## 5. Conclusions

Silvicultural measures to support biodiversity are useful tools for nature protection under the current conditions of intensive human impact on forest ecosystems and ongoing climate change. In the case of the combination of the IAS *Prosopis juliflora* and *Opuntia dillenii*, a dense thicket is created by the former in the upper story and the latter in the under story. At the Ramsar site and Bundala National Park in Sri Lanka, the control variant without any silvicultural measures showed the lowest plant species diversity, gradually decreasing over time. Hard pruning, on the other hand, showed similarly low results. The best results in terms of supporting diversity were reached by various technical measures, including complete uprooting combined with the planting or natural regeneration of native species. Such treatments had substantial positive effects on species diversity indices and negative effects on the number of individuals of *Prosopis juliflora*. The presented results confirm the crucial role of management interventions in areas where IAS are expanding. Therefore, silviculture measures proved to be beneficial tools regarding nature conservation without the use of pesticides at different sites and especially in protected areas to improve habitat diversity.

## Figures and Tables

**Table 1:** Mean number of recorded plant individuals on permanent research plots differentiated according to the treatment (T1–T9) and plant species in 2017–2021 (mean of 9 repeated measurements and 4 replications); the significantly (*p* < 0.05) highest values are in bold.

Treatment	1. *Prosopis juliflora*	2. *Opuntia dillenii*	3. *Salvadora persica*	4. *Lantana camara*	5. *Senna auriculata*	6. *Limonia acidissima*	7. *Bauhinia racemosa*	8. *Azadirachta indica*	9. *Drypetes sepiaria*	10. *Manilkara hexandra*	11. *Randia dumetorrum*	12. *Derris* spp.	13. *Flueggea leucopyrus*	14. *Solanum melongena*	15. *Stachytarpheta indica*	16. *Senna tora*	17. *Achyranthes aspera*	18. *Pongamia pinnata*	19. *Tamarindus indicus*	20. *Chloroxylon swietenia*	21. *Cassia fistula*	22. *Schleichera oleosa*	23. *Madhuca longifolia*	24. *Syzygium cumini*	25. *Vitex altissima*	26. *Terminalia arjuna*	27. *Phyllanthus emblica*	Overall number of Individuals
**T1**	24cd	**324b**	3a	1a	1a	1	2a	1a	1	0	1b	0	1a	1a	2a	0a	0	0	0	1	1a	0	0	0	0	0	0	**363b**
**T2**	26d	**299b**	3a	2a	2a	0	2a	2b	0	0	0a	0	1a	1a	2a	0	0a	5bc	2b	0	2	2	1	3	1	1	1	**358b**
**T3**	23c	**306b**	3ab	2a	3a	0	2a	1a	0	0	1b	0	2b	2a	4a	2a	1b	2a	0	1	1a	0	0	0	0	0	0	**355b**
**T4**	23c	**308b**	3ab	3a	2a	0	2a	2b	0	0	1bc	0	**3c**	4ab	6a	3a	3c	4b	1a	0	2b	2	1	3	1	1	1	**381b**
**T5**	9b	26a	**5d**	13b	17b	0	**5b**	3c	1	0	1c	0	**3c**	**40d**	**36d**	**22d**	**10e**	0	0	0	**3cd**	0	0	1	0	0	0	196a
**T6**	11b	28a	4bc	14b	19b	0	**5b**	3c	1	0	1c	0	**3c**	33c	28c	**20d**	6d	**6c**	**3c**	0	**3d**	2	1	3	1	1	1	198a
**T7**	4a	28a	**4cd**	13b	**24bc**	1	**5b**	2b	1	1	**2d**	0	**3c**	7ab	23bc	14c	5d	5bc	2ab	1	2bc	2	1	3	1	1	1	153a
**T8**	5a	22a	4bc	**18c**	**28c**	1	**5b**	**5d**	1	0	1b	1	2b	10b	17b	8b	6d	0	0	0	**3d**	0	0	0	0	0	0	135a
**T9**	**39e**	**325b**	3a	1a	0a	0	1a	1a	0	0	1b	0	1a	3a	3a	1a	0	0	0	0	1a	0	0	0	0	0	0	**378b**
Test, *p*-value	KW, *p* < 0.001	KW, *p* < 0.001	KW, *p* < 0.001	KW, *p* < 0.001	KW, *p* < 0.001	–	KW, *p* < 0.001	KW, *p* < 0.001	–	–	KW, *p* < 0.001	–	KW, *p* < 0.001	KW, *p* < 0.001	KW, *p* < 0.001	KW, *p* < 0.001	KW, *p* = 0.028	KW, *p* < 0.001	KW, *p* < 0.001	–	KW, *p* < 0.001	–	–	–	–	–	–	KW, *p* < 0.001

**Table 2:** Percentage change (in %) in the number of recorded plant individuals on permanent research plots differentiated according to the treatment (T1–T9) and plant species—comparing year 2017 with 2021. Decrease (negative values is highlighted in red, increase (positive values) is highlighted in green.

Treatment	1. *Prosopis juliflora*	2. *Opuntia dillenii*	3. *Salvadora persica*	4. *Lantana camara*	5. *Senna auriculata*	6. *Limonia acidissima*	7. *Bauhinia racemosa*	8. *Azadirachta indica*	9. *Drypetes sepiaria*	10. *Manilkara hexandra*	11. *Randia dumetorrum*	12. *Derris* spp.	13. *Flueggea leucopyrus*	14. *Solanum melongena*	15. *Stachytarpheta indica*	16. *Senna tora*	17. *Achyranthes aspera*	18. *Pongamia pinnata*	19. *Tamarindus indicus*	20. *Chloroxylon swietenia*	21. *Cassia fistula*	22. *Schleichera oleosa*	23. *Madhuca longifolia*	24. *Syzygium cumini*	25. *Vitex altissima*	26. *Terminalia arjuna*	27. *Phyllanthus emblica*	Total Number of Individuals
**T1**	0	−49,775	0	40	0	0	0	0	0	0	0	↗	0	0	0	0	0	0	0	0	25	0	0	0	0	0	0	−1219
**T2**	0	−59,800	0	33	0	0	0	−14	0	0	0	0	0	0	0	0	↗	−78	−100	0	−60	−14	−33	−9	0	↗	↗	−753
**T3**	38	−180,800	0	63	50	0	10	0	0	0	0	0	0	0	12	0	0	0	0	0	25	0	0	0	0	0	0	−866
**T4**	33	−89,850	0	56	11	0	0	0	0	0	0	0	0	0	0	0	−27	−129	−140	0	−60	0	0	0	0	100	100	−579
**T5**	41	−900	38	57	40	↗	39	27	50	0	0	0	0	2	15	33	−71	0	0	50	36	0	0	75	0	0	0	13
**T6**	35	↙	35	40	35	0	23	42	50	↗	0	0	0	20	8	23	−40	−39	−9	↗	20	0	0	0	0	100	100	9
**T7**	7	−1633	0	7	0	0	0	0	0	33	0	0	0	10	3	0	−50	−88	−100	0	−33	−14	0	0	0	100	100	−20
**T8**	10	−914	0	11	7	0	0	5	25	0	0	0	0	0	7	22	−22	0	0	0	0	0	0	0	0	0	0	−8
**T9**	−1	−19,110	0	0	0	0	0	0	0	0	0	0	0	−8	0	0	0	0	0	0	0	0	0	0	0	0	0	−870

Note: arrows indicate changes compared to the previous year if one value was zero.

**Table 3:** Mean plant species diversity on permanent research plots differentiated according to the treatment (T1–T9) and number of plant species in 2017–2021 (mean of 9 repeated measurements and 4 replications); the significantly (*p* < 0.05) highest values are in bold.

	Number Species		Species Diversity				Species Heterogeneity				Species Evenness			
	m		*D*1		*D*2		Λ		*H*′		*E*1		*E*2	
**T1**	12b	↗	2.046b	↗	0.820bc	↗	0.284a	↗	0.670a	↗	0.282a	↗	0.400a	↗
**T2**	16c	↗	2.661c	↗	1.015cd	↗	0.369ab	↗	0.979bc	↗	0.351a	↗	0.385a	↗
**T3**	12b	↗	1.945b	↗	0.751ab	↗	0.313ab	↗	0.786ab	↗	0.319a	↗	0.404a	↗
**T4**	18d	↗	2.936c	↗	1.054cd	↗	0.392b	↗	1.088c	↗	0.373a	↗	0.371a	↗
**T5**	15c	↗	2.737c	↗	1.104d	↗	**0.844c**	↗	2.192d	↗	**0.800b**	↘	**0.751d**	↘
**T6**	21e	↗	3.718d	↗	1.476e	↗	**0.873c**	↗	**2.461de**	↘	**0.811b**	↘	**0.728d**	↘
**T7**	**23f**	↗	**4.283e**	↗	**1.829f**	↗	**0.867c**	↘	**2.489e**	↗	**0.798b**	↘	0.677c	↘
**T8**	15c	→	2.764c	↗	1.246de	↗	**0.852c**	↘	**2.235de**	↗	**0.836b**	↘	**0.761d**	↘
**T9**	9a	→	1.400a	↗	0.551a	↗	0.288ab	↗	0.635a	↗	0.294a	↗	0.468b	↗
Test	KW, *p* < 0.001		KW, *p* < 0.001		KW, *p* < 0.001		KW, *p* < 0.001		KW, *p* < 0.001		KW, *p* < 0.001		KW, *p* < 0.001	

Notes: arrows indicate value changes in dynamics from 2017 to 2021 (↗—increase; ↘—decrease; →—no changes).

**Table 4:** Nine different variants (their abbreviations are in bold in the first column) of silvicultural treatments used for the desired control of invasive *Prosopis juliflora* trees in permanent research plots in Bundala National Park.

Treatment	Description
**T1**	Hard pruning of *Prosopis juliflora* trees, which allows for regeneration of natural vegetation. This only allows keeping one straight branch or main stem of the tree. Most trees do not have straight stems but have more lateral branches.
**T2**	Hard pruning of *Prosopis juliflora* trees followed by replanting of the chosen indigenous species.
**T3**	Thinning of *Prosopis juliflora* trees, which allows for regeneration of natural vegetation.
**T4**	Thinning of *Prosopis juliflora* trees and the replanting of the chosen indigenous species.
**T5**	Complete uprooting of *Prosopis juliflora* trees, which allows for regeneration of natural vegetation.
**T6**	Complete uprooting of *Prosopis juliflora* trees and replanting of the chosen indigenous species.
**T7**	Replanting of the chosen indigenous species on a *Prosopis juliflora*-free site.
**T8**	Allowing for natural regeneration of vegetation on a *Prosopis juliflora*-free site.
**T9**	Control. Examination of the plot that contains *Prosopis juliflora* without any silvicultural measures.

**Table 5:** Overview of indices describing the plantation diversity and their common interpretations.

Criterion	Reference	Evaluation	Equation
Species diversity	Margalef (1958) [102]	The number of species determined based on the number of plant species on the plot and the number of plants; minimum *D* = 0; higher *D* = higher values.	$D1=\frac{m-1}{\ln(N)}$
	Menhinick (1964) [103]		$D2=\frac{m}{\sqrt{N}}$
Species heterogeneity	Simpson (1949) [104]	The index combining species richness and evenness; calculated based on the number of individual plants; minimum *λ*/*H*′ = 0, higher *λ*/*H*′ = higher values.	$\lambda =1-\sum_{i=1}^{m}w_{i}^{2}$
	Shannon (1948) [105]		$H\prime =-\sum_{i=1}^{m}\left[w_{i}\cdot \ln(w_{i})\right]$
Species evenness	Pielou (1975) [106]	The level of evenness in the representation of individual plant species in the plot; range 0–1; minimum *E* = 0, maximum *E* = 1.	$E1=\frac{H\prime }{\ln(m)}$
	Hill (1973) [107]		$E2=\frac{\frac{1}{1-\lambda }-1}{e^{H\prime }-1}$

Notes: *m* = number of tree species; *N* = number of trees; *w_i_* = proportions of individual plant species; *H*′ = entropy (*H*′) according to Shannon (1948) [105]; *λ* = lambda according to Simpson (1949) [104].

## Data Availability

Source data are available from the authors upon request.

## Supplementary Materials

The following supporting information can be downloaded at https://www.mdpi.com/article/10.3390/plants13010121/s1, Figure S1: Dense bush created by IAS (invasive alien species) *Prosopis juliflora* (Sw.) DC. and the cactus *Opuntia dillenii* (Ker-Gawl.) Haw., which suppresses the natural regeneration of indigenous species. The first author of the study, Channa Suraweera, is in the photo (for scale). Photo: Josef Gallo, 2018/08/11.
