# Supplementary material for: Silvicultural Practices for Diversity Conservation and Invasive Species Suppression in Forest Ecosystems of the Bundala National Park, Sri Lanka
Source: Plants (Basel). 2023 Dec 31;13(1):121. doi: 10.3390/plants13010121 (PMC10780521; doi:10.3390/plants13010121)
Supplement: Supplementary file 1 [file plants-13-00121-s001.zip › plants-2739323-supplementary.pdf]

Supplementary Material

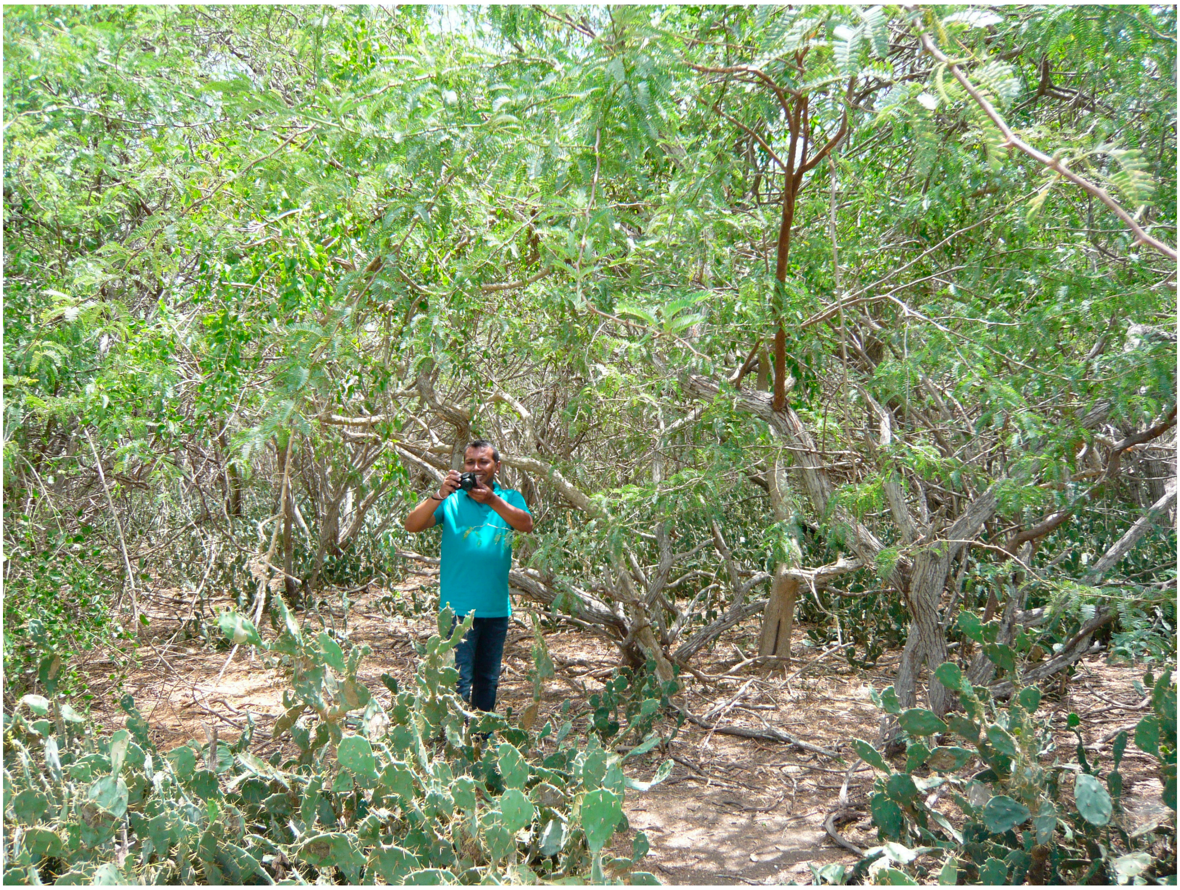

Figure S1. Dense bush created by IAS (invasive aliens species) *Prosopis juliflora* (Sw.) DC., and the cactus *Opuntia dillenii* (Ker-Gawl.) Haw., that suppresses the natural regeneration of indigenous species. First author of the study Channa Suraweera on photo (for scale). Photo: Josef Gallo, 2018/08/11.
